# 

**Published:** 1883-06

**Authors:** 


### EDITORIAL.

### Indiana State Dental Meeting.

The twenty-fifth annual meeting of the Indian State Dental Society will be held in the parlors of the Dennison House, in Indianapolis, beginning on Tuesday, June 26th, and continues its sessions three days and evenings. The subjects for consideration and discussion will be the same as those presented at the first meeting of the body in 1858, viz:

1st. The best means of preserving the teeth.
2nd. Treatment of exposed pulps.
3rd. The best means to correct irregularity of teeth.
4th. Mechanical Dentistry.
5th. Filling teeth.
6th. Miscellaneous subjects.

This will doubtless be a glorious meeting of this grand old society, there will be a large attendance, and an interesting time.

The meeting will close with a jubilee and banquet, on the evening of the last day.

The Dennison, which is the finest hotel in the state will entertain those attending the meeting, at $2.50 per day.

A cordial invitation is extended, and it is hoped there will be a large attendance of the profession from the surrounding states.

---

THE "Illinois State Dental Society" held its annual meeting during the second week of May, at Decatur. The session was one of the most interesting and instructive in its history, and was participated in by a full attendance of active and corresponding members.

The officers elected for the ensuing year are as follows:

President—Edmund Noyes, Chicago; Vice President—W. H. Townsend, Pontiac; Secretary—J. W. Wassall, Chicago; Assistant Sec'y—W. H. Taggart, Freeport; Treasurer—J. Campbell, Bloomington; Librarian—W. B. Woodward, Peoria; Executive Committee—M. H. Patton, Springfield; C. R. Taylor, Streator; C. R. Dwight, Danville.

Board of Examiners—T. L. Gilmer, Quincy; K. B. Davis, Springfield; E. D. Swain, Chicago.

Publication Committee—J. W. Wassall, Chicago; E. D. Swain, Chicago.

Committee on Infraction of the Code of Ethics—P. Neal Lawrence, Lincoln; J. W. Cormany, Mt. Carroll; C. P. Pruyne, Chicago.

### Kentucky State Dental Association.

The annual meeting of this body will be held in the rooms of the Palytechnic Society at Louisville, on Tuesday, June 5th, 1883 at 2½ o'clock p. m. A full attendance of the members is urgently requested, and the profession both in and out of the state, is particularly invited.

Prof. T. W. Tobin, C.E., Ph.D., of the Polytechnic Society will deliver a lecture on the science of cooking food.

The following subjects will be presented for consideration:

1. Anatomy. 2. Physiology. 3. Pathology. 4. Chemistry. 5. Therapeutics. 6. Operative Dentistry. 7. Mechanical Dentistry. There will be papers presented upon each of these subjects, each of which will be discussed.

From the indications, the meeting promises to be largely attended, and one of much interest and profit. Let all contribute by presence, and work to make the meeting a good one.

### Hymenial.

The following marriage announcement, just received, will be of interest to the class of '82 of the University of Michigan. HATTIE L. MARTINDALE, D.D.S., of Grand Rapids, and WILBER O. STUDLEY, D.D.S., of New York, Married, Thursday, May 17th, Grand Rapids, Mich., 1883.

May the greatest success attend this new firm of the Drs. Studley, and it certainly will, if those first happy months of their acquaintance in Ann Arbor, are to be omens for all the years to come in their united lives. H.

### Removal.

Dr. E. G. Betty, D.D.S., has removed his residence and office to No. 334 Race St., third door north of Ninth Street, Cincinnati. May Edward reap all that is usual upon a removal to fresh pastures.

### CONTENTS.

### COMMUNICATIONS.

A MONTHLY MAGAZINE DEVOTED TO THE INTERESTS OF THE

### DENTAL PROFESSION.

*Terms Reduced to $2.00 Per Year. Single Copies, 25 Cents.*

### EDITED BY PROF. J. TAFT, D.D.S.

—AND—

*Published by SPENCER & CROCKER, Ohio Dental Depot.*

The volume commences in January and closes in December.

The REGISTER being issued monthly is always fresh, therefore Indispensable to the practitioner who desires to keep posted up with the new inventions and improvements which are daily developed. Neither expense nor effort will be spared to give value and variety to its contents. Articles will be illustrated with well executed engravings whenever they will add to the interest or assist to explanation.

Its extensive circulation and frequency of issue make it one of the BEST DENTAL ADVERTISING MEDIUMS in the United States.

All subscriptions must begin with January or July.

Local or special notices, five lines or less, will be inserted for $3 each insertion; over five lines, 50 cts. per line.

All advertising bills payable in advance, or at furthest, after the issue of the first number containing the advertisement. All transient advertisements to be paid for in advance.

☞CONTRIBUTIONS SOLICITED.

Exclusively Editorial Communications should be addressed to the Editor.

The latest number of the REGISTER may be ordered with or without other goods at any time, and all letters should be addressed to

**SPENCER & CROCKER, Ohio Dental Depot, Cincinnati, O.**

